# Association Between Carpal Tunnel Syndrome and Subsequent Heart Failure Among Adults in Germany

**DOI:** 10.1001/jamanetworkopen.2023.23091

**Published:** 2023-07-12

**Authors:** Mark Luedde, Volker Jürgen Schmidt, Julia Gänsbacher-Kunzendorf, Karel Kostev

**Affiliations:** 1 Christian-Albrechts-University of Kiel, Kiel, Germany; 2 Cardiology Joint Practice, Bremerhaven, Germany; 3 Department of Hand, Reconstructive and Plastic Surgery, Kantonspital St Gallen, St Gallen, Switzerland; 4 University of Heidelberg, Heidelberg, Germany; 5 Cardiology, University Medical Center of Schleswig Holstein, Campus Kiel, Kiel, Germany; 6 Epidemiology, IQVIA, Frankfurt, Germany

## Abstract

This cohort study investigates the association of carpal tunnel syndrome with heart failure among a large group of outpatients in Germany.

## Introduction

Carpal tunnel syndrome (CTS), a common nerve compression syndrome, is one of the most common causes of hand surgery.^1^ Recently, CTS has been associated with to heart failure (HF), mainly through an increased rate of detection of transthyretin (ATTR) cardiac amyloidosis among patients with CTS.^2^ We investigate the association of CTS with HF among a large collective of outpatients.

## Methods

This retrospective cohort study was based on data from the Disease Analyzer database (IQVIA). Adult patients (aged ≥18 years) from 1284 general practices in Germany between January 1, 2005, and December 31, 2020, with an initial diagnosis of CTS were included. Our protocol was evaluated by the local ethics committee of Christian-Albrechts-University of Kiel. Because we used only anonymized data, it was not necessary to obtain informed consent from individual patients (eMethods in Supplement 1). This study followed the Strengthening the Reporting of Observational Studies in Epidemiology (STROBE) reporting guideline.

Individuals without CTS were matched to patients with CTS using propensity score matching (1:1). The main outcome was the initial diagnosis of HF up to 10 years after the index date. As a negative control, we analyzed the association of CTS with cancer. Because of the high patient count and multiple comparisons, a 2-sided *P* < .001 was considered statistically significant.

## Results

### Basic Characteristics

The study included 81 898 patients with CTS (mean [SD] age, 52.9 [15.1] years; 54 626 women [66.7%]) and 81 898 without CTS (mean [SD] age, 52.7 [15.3] years; 54 298 women [66.3%]). The characteristics are displayed in the Table. Patients visited their physicians a mean (SD) 8.2 (6.4) times per year during follow-up. Within 12 months prior to the index date, there were no differences between study cohorts in predefined diagnoses and therapies.

**Table.** Baseline Characteristics of the Study Sample (After Propensity Score Matching)

Variable	Patients, No. (%)	
	CTS (n = 81 898)	No CTS (n = 81 898)
Age, y		
Mean (SD)	52.9 (15.1)	52.7 (15.3)
18-40	17 444 (21.3)	18 018 (22.0)
41-50	18 263 (22.3)	17 936 (21.9)
51-60	22 522 (27.5)	22 276 (27.2)
61-70	11 630 (14.2)	11 630 (14.2)
>70	12 039 (14.7)	12 039 (14.7)
Gender		
Women	54 626 (66.7)	54 298 (66.3)
Men	27 272 (33.3)	27 600 (33.7)
No. of physician visits/y during follow-up, mean (SD)	8.2 (6.4)	8.2 (6.4)
Diabetes	12 448 (15.2)	12 367 (15.1)
Obesity	8108 (9.9)	7944 (9.7)
Lipid metabolism disorders	21 867 (26.7)	21 539 (26.3)
Hypertension	31 121 (38.0)	30 957 (37.8)
Chronic bronchitis or COPD	6224 (7.6)	6060 (7.4)
Drug classes prescribed ≤12 mo prior to the index date		
Diuretics	10 647 (13.0)	10 811 (13.2)
β-Blockers	11 548 (14.1)	12 776 (15.6)
Calcium channel blockers	5897 (7.2)	6634 (8.1)
ACE inhibitors	11 056 (13.5)	11 957 (14.6)
Angiotensin II receptor blockers	8517 (10.4)	8517 (10.4)
Statins	6879 (8.4)	7944 (9.7)

Abbreviations: ACE, angiotensin-converting enzyme; COPD, chronic obstructive pulmonary disease.

### Incidence of HF

Ten years after the index date, 8.4% of patients with CTS and 6.2% of those without CTS received a diagnosis of HF (*P* < .001) (Figure). The incidence was 8.7 vs 6.1 cases per 1000 patient years.

**Figure.**
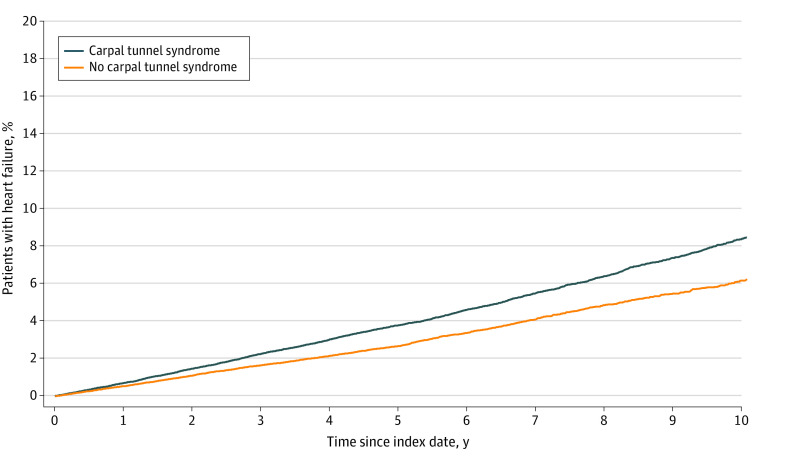
Cumulative Incidence of Heart Failure Among Patients With or Without Carpal Tunnel Syndrome

### Association of CTS With Subsequent HF and Amyloidosis

In the regression analysis, there was a significant association between CTS and subsequent HF diagnosis (hazard ratio [HR], 1.39; 95% CI, 1.31-1.47). This association was similar between women (HR, 1.40; 95% CI, 1.30-1.50) and men (HR, 1.38; 95% CI, 1.25-1.52). However, while CTS was associated with HF among patients aged 61 to 70 years (HR, 1.48; 95% CI, 1.35-1.61) and more than 70 years (HR, 1.48; 95% CI, 1.35-1.61), no significant associations were observed among patients aged 18 to 40 years, 41 to 50 years, or 51 to 60 years.

A total of 47 patients in the CTS group and 17 patients in the control group received a diagnosis of amyloidosis. The adjusted HR for amyloidosis in the CTS group was 1.79 (95% CI, 1.01-3.18; *P* = .05). A sensitivity analysis using a multivariate regression model provided an HR of 1.24 (95% CI, 1.20-1.29) for HF among the CTS group. As a negative control, there was no association between CTS and cancer (HR, 1.04; 95% CI, 0.87-1.09; *P* = .17).

## Discussion

This study found that CTS was significantly associated with an increased incidence of new-onset HF. The increased rate of HF among patients with CTS requires attention because HF is a common disease associated with high mortality.^2,3^ Early diagnosis of HF is a key to successful treatment,^3^ particularly for ATTR cardiac amyloidosis, which has been associated with CTS in a recent study.^2^ The importance of cardiac amyloidosis for HF with preserved ejection fraction has been demonstrated.^4^

Our study has some limitations. Data are based on *ICD-10* codes only. The absolute number of patients with amyloidosis recorded in our study is relatively small, there is uncertainty about the proportion of patients with ATTR cardiac amyloidosis, and underrecording is suspected. Because our database does not offer data on the severity of cardiovascular disease, we cannot ensure that both groups are balanced in this regard.

However, our study reveals an association between CTS and HF. Thus, we point to a new role for surgeons together with general practitioners in the early detection of important internal diseases, a chance for better treatment, and an improved prognosis of these diseases.

## Notes

Supplement 1.
eMethods.

eReferences.

Supplement 2.
Data Sharing Statement
