# Supplementary material for: Association Between Carpal Tunnel Syndrome and Subsequent Heart Failure Among Adults in Germany
Source: JAMA Netw Open. 2023 Jul 12;6(7):e2323091. doi: 10.1001/jamanetworkopen.2023.23091 (PMC10339157; doi:10.1001/jamanetworkopen.2023.23091)
Supplement: Supplement 1. — eMethods. eReferences. [file jamanetwopen-e2323091-s001.pdf]

Supplementary Online Content

Luedde M, Schmidt VJ, Gänsbacher-Kunzendorf J, Kostev K. Association between carpal tunnel syndrome and subsequent heart failure among adults in Germany. *JAMA Netw Open.* 2023;6(7):e2323091. doi:10.1001/jamanetworkopen.2023.23091

eMethods.
eReferences.

This supplementary material has been provided by the authors to give readers additional information about their work.

© 2023 Luedde M et al. *JAMA Network Open.*

eMethods.

Database

This retrospective cohort study was based on data from the Disease Analyzer database (IQVIA), which contains drug prescriptions, diagnoses, and basic medical and demographic data obtained directly and in anonymous format from computer systems used in the practices of general practitioners and specialists[1]. The database covers approximately 3% of all private practices in Germany. The sampling method for the Disease Analyzer database is based on summary statistics from all doctors in Germany published yearly by the German Medical Association. IQVIA uses these statistics to determine the panel design according to the four strata including specialist group, German federal state, community size category, and age of physician. It has previously been shown that the panel of practices included in the Disease Analyzer database is representative of general and specialized practices in Germany[1]. Finally, this database has already been used in previous studies focusing on cardiovascular disorders[2,3].

Study population

This study included adult patients (≥18 years) with an initial diagnosis of carpal tunnel syndrome (CTS) (ICD-10: G56.0) in 1,284 general practices in Germany between January 2005 and December 2020 (index date; Supplementary Figure 1). Further inclusion parameter was an observation time of at least 12 months prior to the index date and a follow-up time of at least six months after the index date. Patients with diagnoses of ischemic heart disease (ICD-10: I20-I25), atrial fibrillation (I48.0,I48.1,I48.2,I48.9), or heart failure (ICD-10: I50) prior to or on the index date were excluded.

After applying similar inclusion criteria, individuals without CTS were matched to CTS patients using propensity score matching (1:1) based on sex, age, yearly consultation frequency during the follow-up, diabetes, obesity, lipid metabolism disorders, hypertension and chronic bronchitis/chronic obstructive pulmonary disease (COPD) diagnoses prior to the index date.

© 2023 Luedde M et al. *JAMA Network Open.*

For the non-CTS cohort, the index date was that of a randomly selected visit between January 2005 and December 2020 (Figure 1). Diabetes (ICD-10: E10-E14), obesity (ICD-10: E66), lipid metabolism disorders (ICD-10: E78) and hypertension (ICD-10: I10) were included as these diagnoses are strongly associated with heart diseases. As the database used does not contain information on smoking status, chronic bronchitis (ICD-10: J42) and COPD (ICD-10: J44) diagnoses were used in matching due to their strong association with smoking behavior[4]. Propensity score matching balance was considered to be achieved when the difference between proportions of co-variables was not higher than 1%.

Study outcomes and statistical analyses

The outcomes of the study were the initial diagnoses of heart failure (HF) in the up to 10 years following the index date as function of CTS. Additionally, the initial diagnoses of amyloidosis (ICD-10: E55) in the up to 10 years following the index date as function of CTS were evaluated. As a negative control, we analyzed the association of CTS with cancer (ICD-10: C00-C97).

Differences in the sample characteristics and diagnosis prevalence between CTS and non-CTS cohorts were compared using the Wilcoxon signed-rank test for continuous variables, the McNemar test for categorical variables with two categories, and the Stuart-Maxwell test for categorical variables with more than two categories.

The 10-year cumulative incidence of HF and amyloidosis in the cohort with and without CTS was further studied with Kaplan-Meier curves, and these curves were compared using the log-rank test. Incidence was calculated as case per 1000 patient-years. Finally, a Cox regression analysis adjusted for cardiovascular drug prescriptions was conducted to assess the association between CTS and HF. These prescriptions documented in the 12 months prior to or at the index date included diuretics, beta blockers, calcium channel blockers, ACE inhibitors, Angiotensin II receptor blockers, and statins. Results of the Cox regression model are displayed as hazard ratios (HRs) and 95% confidence intervals (CIs). Additionally, Cox regression analyses were

© 2023 Luedde M et al. *JAMA Network Open.*

conducted separately for women and men, and five age groups. As sensitivity analyses, multivariable regression models were additionally conducted for unmatched cohorts adjusted for sex, age, yearly consultation frequency during the follow-up, diabetes, obesity, lipid metabolism disorders, hypertension, chronic bronchitis/ COPD diagnoses prior to the index date, and cardiovascular drug prescriptions. Due to high patient count and multiple comparisons p-value of <0.001 was considered statistically significant. Two-sided p-values were used. Analyses were carried out using SAS version 9.4 (SAS Institute, Cary, USA). Microsoft excel was used to prepare Figure 1. The guidelines put up in the "Strengthening the Reporting of Observational Studies in Epidemiology (STROBE) Statement (https://www.equator-network.org/reporting-guidelines/strobe/)" were followed in the documentation of our cohort study and the preparation of the manuscript.

Ethical standards

Only aggregated, anonymized patient data were used in these analyses. This study was performed in accordance with the Declaration of Helsinki and the Good Practice of Secondary Data Analysis guidelines[5]. Our Research protocol was evaluated by the local ethics committee of Christian-Albrechts-University (CAU) Kiel, Germany: Since we used only anonymized data, which could not be traced back to individual persons, it was confirmed that it was not necessary to obtain informed consent from individual patients to participate in the study (File reference D413/21 of the CAU ethics committee).

© 2023 Luedde M et al. *JAMA Network Open.*

eReferences.

1. Rathmann W, Bongaerts B, Carius HJ, Kruppert S, Kostev K. Basic characteristics and representativeness of the German Disease Analyzer database. *Int J Clin Pharmacol Ther.* 2018;56(10):459-466.
2. Loosen SH, Roderburg C, Jahn JK, et al. Heart failure and depression: a comparative analysis with different types of cancer. *Eur J Prev Cardiol.* 2021.
3. Roderburg C, Loosen SH, Jahn JK, et al. Heart failure is associated with an increased incidence of cancer diagnoses. *ESC Heart Fail.* 2021;8(5):3628-3633.
4. Pelkonen M. Smoking: relationship to chronic bronchitis, chronic obstructive pulmonary disease and mortality. *Curr Opin Pulm Med.* 2008;14(2):105-109.
5. Swart E, Gothe H, Geyer S, et al. [Good Practice of Secondary Data Analysis (GPS): guidelines and recommendations]. *Gesundheitswesen.* 2015;77(2):120-126.

© 2023 Luedde M et al. *JAMA Network Open.*
