# Supplementary material for: Association Between Carpal Tunnel Syndrome and Subsequent Heart Failure Among Adults in Germany
Source: JAMA Netw Open. 2023 Jul 12;6(7):e2323091. doi: 10.1001/jamanetworkopen.2023.23091 (PMC10339157; doi:10.1001/jamanetworkopen.2023.23091)
Supplement: Supplement 2. — Data Sharing Statement [file jamanetwopen-e2323091-s002.pdf]

Data Sharing Statement

Luedde. Association Between Carpal Tunnel Syndrome and Subsequent Heart Failure Among Adults in Germany. *JAMA Netw Open*. Published July 12, 2023.
doi:10.1001/jamanetworkopen.2023.23091

Data
Data available: Yes
Data types: Deidentified participant data
How to access data: on reasonable request
When available: With publication

Supporting Documents
Document types: None

Additional Information
Who can access the data: anyone with reasonable request
Types of analyses: for comprehension
Mechanisms of data availability: with signed agreement
